# Multiple *Orientia tsutsugamushi* Ankyrin Repeat Proteins Interact with SCF1 Ubiquitin Ligase Complex and Eukaryotic Elongation Factor 1 α

**DOI:** 10.1371/journal.pone.0105652

**Published:** 2014-08-28

**Authors:** Chan-Ki Min, Ye-Jin Kwon, Na-Young Ha, Bon-A Cho, Jo-Min Kim, Eun-Kyung Kwon, Yeon-Sook Kim, Myung-Sik Choi, Ik-Sang Kim, Nam-Hyuk Cho

**Affiliations:** 1 Department of Microbiology and Immunology, Seoul National University College of Medicine, Seoul, Republic of Korea; 2 Department of Biomedical Science, Seoul National University College of Medicine, Seoul, Republic of Korea; 3 Divisions of Infectious Diseases, Chungnam National University Hospital, Daejeon, Republic of Korea; 4 Institute of Endemic Disease, Seoul National University Medical Research Center and Bundang Hospital, Seoul, Republic of Korea; University of Louisville, United States of America

## Abstract

**Background:**

*Orientia tsutsugamushi*, the causative agent of scrub typhus, is an obligate intracellular bacterium. Previously, a large number of genes that encode proteins containing eukaryotic protein-protein interaction motifs such as ankyrin-repeat (Ank) domains were identified in the *O. tsutsugamushi* genome. However, little is known about the Ank protein function in *O. tsutsugamushi.*

**Methodology/Principal Findings:**

To characterize the function of Ank proteins, we investigated a group of Ank proteins containing an F-box–like domain in the C-terminus in addition to the Ank domains. All nine selected *ank* genes were expressed at the transcriptional level in host cells infected with *O. tsutsugamushi*, and specific antibody responses against three Ank proteins were detected in the serum from human patients, indicating an active expression of the bacterial Ank proteins post infection. When ectopically expressed in HeLa cells, the Ank proteins of *O. tsutsugamushi* were consistently found in the nucleus and/or cytoplasm. In GST pull-down assays, multiple Ank proteins specifically interacted with Cullin1 and Skp1, core components of the SCF1 ubiquitin ligase complex, as well as the eukaryotic elongation factor 1 α (EF1α). Moreover, one Ank protein co-localized with the identified host targets and induced downregulation of EF1α potentially via enhanced ubiquitination. The downregulation of EF1α was observed consistently in diverse host cell types infected with *O. tsutsugamushi*.

**Conclusion/Significance:**

These results suggest that conserved targeting and subsequent degradation of EF1α by multiple *O. tsutsugamushi* Ank proteins could be a novel bacterial strategy for replication and/or pathogenesis during mammalian host infection.

## Introduction

*Orientia tsutsugamushi*, the causative agent of scrub typhus endemic to the Asia-Pacific region, is a mite-borne, obligate intracellular bacterium [1]. Scrub typhus is an acute febrile illness characterized by fever, rash and eschar, and often leads to severe clinical complications such as interstitial pneumonia, acute renal failure, meningoencephalitis, gastrointestinal bleeding, and multi-organ failure [2], [3]. An estimated one billion people in the endemic area are at risk for scrub typhus and an estimated one million new cases occur annually [2]. Scrub typhus can be effectively treated with antibiotics including doxycycline, chloramphenicol, and azithromycin. However, re-infection is common due to the wide variety of antigenically distinct serotypes [4]. In addition, the increasing number of patients, recurrent outbreaks, and new emergences of scrub typhus within endemic areas have recently had a profound effect on public health [5]–[8].

As an obligate intracellular pathogen, *O. tsutsugamushi* may have evolved mechanisms that enable it to survive within eukaryotic host cells and overcome the defense systems of the host. Symbiotic maintenance of *O. tsutsugamushi* in trombiculid mites by transovarian transmission may have resulted from longstanding associations with the arthropod host and genomic specialization to an intracellular lifestyle [9], [10]. Accidental transfer of *O. tsutsugamushi* to mammalian hosts during the mite feeding process can result in inflammatory disease, the severity of which correlates with bacterial burden in systemic circulation [11]. This finding suggests that bacterial evasion of the mammalian immune system and subsequent replication might be crucial determinants for bacterial pathogenicity. However, understanding of bacterial survival strategies and pathogenesis within eukaryotic host systems has been limited by the scarce information on bacterial virulence factors and lack of methodology for genetic modification. Therefore, previous studies have largely focused on the bacterial interaction with the immune cells of the host, surface receptors, and/or signaling pathways to elucidate mechanisms underlying the inflammation and invasion processes [12]–[21]. After the complete genomic sequencing of *O. tsutsugamushi* [22], [23], functional genomic analysis and global gene expression profiling of the intracellular pathogen have provided valuable insights into the bacterial adaptation to eukaryotic host systems in terms of genomic evolution, metabolic capability, and novel bacterial virulence [24]–[28].

Among the potential virulence genes identified in the *O. tsutsugamushi* genome, which are of particular interest for host-parasite interactions, there are many genes encoding proteins containing eukaryotic protein-protein interaction motifs such as tetratricopeptide-repeat or ankyrin-repeat (Ank) domains [22]. Ank proteins contain a tandem motif of approximately 33 amino acids, one of the most common modular protein-protein interaction motifs in eukaryotes [29]. Genes encoding proteins with these domains, which are generally rare in prokaryotes, are thought to have arisen by interdomain horizontal gene transfer from an eukaryotic source [30]. Functional mimicry of eukaryotic protein activity by a bacterial protein family is a well-established virulence mechanism used to manipulate a variety of host cell activities at the molecular level [31], [32]. The presence of eukaryotic-like proteins has also been reported in diverse facultative or obligate intracellular bacteria including *Legionella pneumophila* [30], *Coxiella burnetii* [33], *Anaplasma phagocytophilum* [34], *Wolbachia* spp. [35], [36], and *Rickettsia* spp. [37]. In addition, increasing evidence indicates that bacterial Ank proteins are bona fide effectors of the type 1 secretion system (T1SS) [38], [39] or type 4 secretion system (T4SS), which deliver them into host cells to regulate host processes important for bacterial survival or replication [40], [41]. Two ancestral lineages for the T4SS are currently known: the *virB*/*virD* system of *Agrobacterium tumefaciens* and the *dot*/*icm* system of *Legionella pneumophila*, referred to as T4aSS and T4bSS, respectively [42], [43]. Like in other members of the Rickettsiales order, the *O. tsutsugamushi* genome encodes protein secretion systems [22], [42]. Phylogenomic analysis of T4SS detected in members of the Rickettsiales order revealed that they are homologous to the T4aSS of *A. tumefaciens* [43]. Thus, after secretion, Ank proteins may function as effectors in bacterial pathogenesis to facilitate host-cell infection and disease progression.

Here, we characterized *O. tsutsugamushi’s* Ank proteins by bioinformatic analysis, examined the subcellular localization of Ank proteins when expressed ectopically in mammalian cells and screened for host targets of the bacterial protein family using GST pull-down assays. Multiple Ank proteins were found to interact with Cullin1 and Skp1, components of the SCF1 ubiquitin ligase complex, and eukaryotic elongation factor 1a (EF1α); one also induced downregulation of EF1α potentially via enhanced ubiquitination of the host proteins. These results may provide valuable clues about the mechanisms of action of Ank proteins, which might be functionally redundant in modulating host processes.

## Materials and Methods

### Ethics statements

Ethical issues in this experiment were approved by the Institutional Review Board of both Seoul National University Hospital (IRB No. 0-1001-039-307) and Chungnam National University Hospital (IRB No. 2008-10-08). All adult patients and healthy volunteers provided written informed consent prior to sample collection.

### Sequence alignment and domain prediction

A*nk* gene sequences from the genomes of the *O. tsutsugamushi* strain Boryong (GenBank ID: AM494475.1) were extracted and aligned for comparison using ClustalW embedded within DNAstar software (DNAstar Inc., Madison, WI). The degree of identity and divergence among the Ank sequences was determined using the calculated alignment. The identification and annotation of protein domains were performed using SMART (Simple Modular Architecture Research Tool, http://smart.embl-heidelberg.de/) program [44].

#### Cell culture

HeLa cells (American Type Culture Collection (ATCC) CCL-2), L929 cells (ATCC NCTC929), and ECV304, an endothelial-like cell line [45], were maintained in DMEM medium (Welgene, Daegu, Korea) supplemented with 10% heat-inactivated fetal bovine serum (FBS) (Welgene), 100 U/mL penicillin, and 100 µg/mL streptomycin (Gibco BRL, Gaithersburg, MD) at 37°C in 5% CO_2_. THP-1 cells were cultured in RPMI-1640 medium (Welgene) supplemented as described above. HMEC-1, derived from human dermal microvascular endothelial cells [13], were propagated in MCDB 131 medium (Life Technologies, Grand Island, NY) supplemented with 15% FBS, 1 mg/ml hydrocortisone (Sigma Chemical Co.), 10 ng/ml epidermal growth factor (Life Technologies), 100 U/mL penicillin, and 100 µg/mL streptomycin. To inhibit proteasome activity, cells were treated with MG132 (carbobenzoxy-Leu-Leu-leucinal, 10 µM, Sigam) for 4 h before harvesting the cells.

### *O. tsutsugamushi*

The *O. tsutsugamushi* Boryong strain was purified using a modified Percoll gradient purification method [19], [46]. *O. tsutsugamushi* was propagated in L929 cells. At 3–4 days post-infection, infectivity was determined by an indirect immunofluorescence assay (IFA, see below). When an infection rate >90% was achieved, cells were harvested by centrifugation at 6000×*g* for 20 min. The cell pellet was resuspended in 6.5 mL Tris-sucrose (TS) buffer (33 mM Tris-Cl (pH 7.4) and 0.25 M sucrose). Resuspended cells were homogenized with a Polytron homogenizer (Wheaton Inc., Millville, NJ) for 100 strokes and centrifuged at 200×*g* for 5 min. The supernatant was mixed with 40% Percoll (Pharmacia Fine Chemicals, Uppsala, Sweden) in TS buffer and centrifuged at 25,000×*g* for 60 min. The bacterial band was collected and centrifuged at 77,000×*g* for 30 min. *O. tsutsugamushi* were collected and washed 3 times with TS buffer. The *O. tsutsugamushi* pellet was resuspended in DMEM and stored in liquid nitrogen until use. The infectivity titer of the inoculum was determined as described [12], with minor modifications. Infected-cell–counting units (icu) were calculated as follows: [(total number of cells used for infection)×(percentage of infected cells)×(dilution of the *O. tsutsugamushi* suspension)]/100 [12]. For infection assays, 1.0×10^7^ icu of *O. tsutsugamushi* were used to infect cells cultured in 6-well plates containing 1.0×10^6^ of host cells.

### Antibodies and serum

Anti-Cullin1 and anti-glyceraldehyde 3-phosphate dehydrogenase (GAPDH) antibodies were purchased from Calbiochem (La Jolla, CA). Anti-EF1α, anti-Skp1, and anti-ubiquitin antibodies were obtained from Millipore (Billerica, MA). The Anti-flag tag monoclonal antibody was bought from Sigma chemicals (St. Louis, MO). HRP-conjugated anti-mouse and anti-rabbit IgG antibodies for immunoblot assays were obtained from Santa Cruz Biotech Inc. (Santa Cruz, CA). AlexaFluor488- and AlexaFluor594-conjuagted anti-mouse and anti-rabbit IgG antibodies were purchased from Molecular Probes (Invitrogen) for immunofluorescence assays. Human pooled sera were prepared from 10 scrub typhus patients (IFA titer≥1∶1280) and 10 healthy volunteers following institutional review board approval and the receipt of informed consent from all subjects.

### Reverse transcription-PCR

To detect *O. tsutsugamushi ank* gene expression, total RNA was isolated from L929 cells infected with *O. tsutsugamushi*. A RNeasy Mini kit (Qiagen, Hilden, Germany) was used to isolate total RNA from infected L929 cells. To remove contaminated DNA, RNA samples were digested with RNase-free DNase (Qiagen) at room temperature. RT-PCR was performed using the Reverse Transcription System (Promega, Madison, WI) and Accupower Pfu PCR kit (Bioneer, Daejon, Korea). The reactions were performed according to the manufacturers’ instructions using 1 µg total RNA and 10 µM of each primer listed in Table S1.

### Gene cloning, purification of GST fusion proteins, and *in vitro* GST pull-down assay

Genes encoding ankyrin repeat proteins were amplified from the genomic DNA of *O. tsutsugamushi* by PCR using the primer sets listed in Table S1. Amplified gene products were cloned into restriction enzyme-digested pGEX4T-1 (GE Healthcare, Piscataway, NJ), or p3xFlagCMV10 (Sigma-Aldrich). All plasmids encoding GST fusion proteins were sequenced to confirm in-frame cloning. Recombinant GST-Ank fusion proteins were produced in *E. coli* BL21 (DE3) harboring a pGEX4T-1 vector encoding the GST fusion protein of interest. Following induction with IPTG (0.5 mM) at 37°C for 4 h or 16°C overnight, the proteins were purified using glutathione-Sepharose 4B columns according to the manufacturer’s instructions (GE Healthcare). Finally, the identity and purity of the purified proteins were assessed by immunoblot analysis and Coomassie blue staining, respectively. The GST pull-down experiments were carried out using the cell lysate of 1×10^8^ ECV304 cells mixed with 20 µg GST or GST-Ank fusion proteins. After incubation for 4 h at 4°C, the Sepharose beads containing GST fusion proteins were washed four times with lysis buffer (0.15 M NaCl, 0.5% Nonidet P-40, 50 mM Tris, pH 7.5) containing protease inhibitor cocktail (Roche, Mannheim, Germany) and subjected to SDS-PAGE followed by peptide sequencing (ProteomeTech Inc., Seoul, Korea) or immunoblotting with antibodies.

### Immunofluorescence microscopy

Immunofluorescence techniques were used to visualize *O. tsutsugamushi* [19]. Infected monolayers of L929 cells on 24-well tissue culture plates were collected by trypsinization at 1 h post-infection. Extracellular or surface-bound bacteria were removed by washing 3 times with PBS. The infected cells were then fixed in cold 100% methanol for 15 min at 4°C and stained as described previously [12]. Cells infected with *O. tsutsugamushi* were incubated with anti-TSA56 [19] followed by Alexa488-conjugated rabbit anti-mouse IgG (Molecular Probes). To assess the co-localization of ankyrin repeat proteins with host proteins, HeLa cells grown on 12-mm-diameter glass coverslips were transfected with plasmids encoding Flag-tagged ankyrin repeat proteins using Lipofectamine 2000 (Invitrogen) according to the manufacturer’s instructions. Two days later, the cells were fixed in PBS containing 4% paraformaldehyde for 15 min at room temperature and permeabilized in 0.2% Triton X-100 for 15 min. Subsequently, the cells were incubated with anti-Cullin1 antibody and anti-Flag antibody for 1 h, followed by AlexaFluor488-conjugated goat anti-mouse IgG and AlexaFluor594-conjugated goat anti-rabbit IgG. The cells were examined with an Olympus FV1000 laser scanning confocal microscope (Olympus; Tokyo, Japan). Images were analyzed and processed using the Olympus Fluoview software or Adobe Photoshop software.

### *In vitro* ubiquitination assay

*In vitro* ubiquitination assay was performed according to the instructions provided with the ubiquitin conjugation kit (HeLa lysate-based; BioMol, Plymouth Meeting, PA). Briefly, the assays were carried out at 37°C for 4 h in the presence or absence of purified GST-Ank1U5 protein at the indicated amounts. To detect the ubiquitination of EF1α, the reaction mixture was analyzed by immunoprecipitation with an anti-EF1α antibody and subsequent immunoblotting with an anti-ubiquitin antibody.

### Real-time RT-PCR

Total RNAs were isolated using TRIzol reagent (Invitrogen) and used for cDNA synthesis (BD Clontech) according to the manufacturers’ instructions. A real-time RT-PCR was performed with a 7500 Real Time PCR system (ABI, Foster City, CA) with 1 µl cDNA and 0.25 µM of each primer (Table S1). The relative amount of EF1α transcripts was calculated after normalization with β-actin transcript [47].

## Results

### Presence of an F-box–like domain in the C-terminal region of *O. tsutsugamushi* ankyrin-repeat proteins

**Figure 1:**
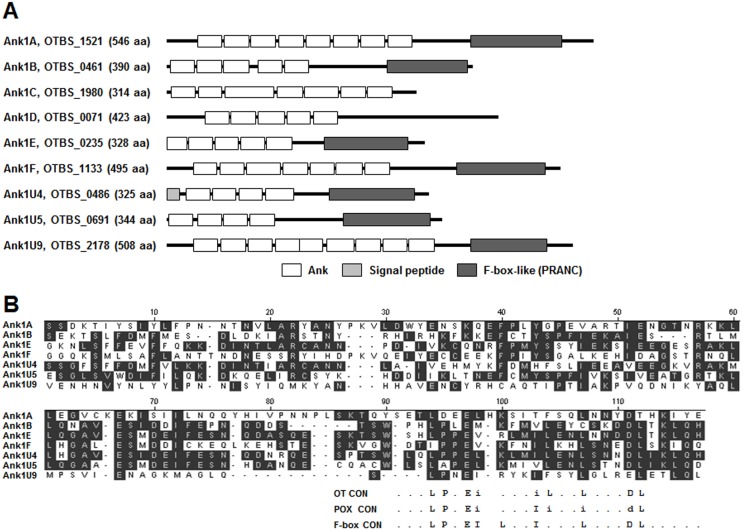
Analysis of *O. tsutsugamushi* Ank proteins. (A) Schematic representation of nine Ank proteins selected from the master genes (Ank1A–F) of 6 subgroups and single-copy genes (Ank1U4, 5, and 9) containing a PRNAC domain in their C-terminus. Each Ank domain predicted by SMART (Simple Modular Architecture Research Tool, http://smart.embl-heidelberg.de/) is shown as a white box at its relative amino acid position within the full length Ank protein. Predicted PRANC domains are indicated by dark gray boxes and signal peptides by a light gray box. (B) Alignment of the amino acid sequences of the C-terminal PRNAC domains of seven Ank proteins. Residues shared by the majority of the seven sequences are shaded. The bottom portion of the figure shows consensus sequences of the F-box (F-box CON) [48] together with F-box–like domains of poxviruses (POX CON) and *O. tsutsugamushi* (OT CON), with lower case letters representing variant positions as follows: i representing I, L or V, and d representing D or E. A dot (.) indicates non-conserved positions.

Previously, 50 ankyrin-repeat (Ank) proteins and fragments thereof were identified in the *O. tsutsugamushi* genome [22]. All ankyrin-repeat genes were flanked on one or both sides by mobile genetic elements such as *tra* genes, *tra*-associated genes, transposases, and/or reverse transcriptases. Based on phylogeny, gene order, and domain structure, 40 Ank proteins formed three broad groups (Ank1), and the remaining 10 single-copy genes (Ank2) showed a loose association with these groups [22]. The 40 Ank proteins (Ank1) were further divided into six subgroups (Ank1A–F), each containing 2–16 members, and nine single-copy genes (Ank1U) after comparison of sequence similarity [22]. The six subgroups share a defined gene order, and each consisted of one full-length “master” gene and several additional nearly identical genes or pseudogene copies. Amino acid sequence alignment of the Ank proteins revealed a C-terminal motif shared by many of the master proteins. In addition to Ank domains, searches of protein domain databases using low cut-off settings detected an F-box–like motif in the C-terminal region of several Ank proteins (Fig. 1A). The F-box–like domain, also known as a PRANC (*p*ox protein *r*epeats of *a*nkyrin *C* terminus) motif, was initially found in the majority of vertebrate poxviruses Ank proteins [48]. Manual inspection of *O. tsutsugamushi* Ank proteins revealed the presence of this PRANC domain in the master genes of four subgroups (Ank1A, Ank1B, Ank1E, and Ank1F) and three single-copy genes (Ank1U4, Ank1U5, and Ank1U9), as shown in Fig. 1A. These seven Ank proteins display only limited overall amino acid sequence identity (13–43%) to each other but share a bipartite, Ank-PRANC domain organization, which has also been reported in other poxviral Ank proteins [48], [49]. In addition, the *O. tsutsugamushi* PRANC domains contained conserved poxviral F-box–like motif amino acids in their C-termini (Fig. 1B), although amino acid sequence identity between oriential and poxviral PRANC domains was quite low (2–20%) when locally aligned. The oriential and poxviral F-box motif is shorter than the typical cellular F-box and is located at the C-terminus of each Ank protein, whereas eukaryotic cellular F-boxes are typically located in the N-terminal region [49]. Based on these structural and sequence characteristics, we selected the six master Ank proteins of each subgroup (Ank1A, 1B, 1C, 1D, 1E, 1F) and three single-copy proteins (Ank1U4, Ank1U5, Ank1U9) that contained the F-box–like domain (Fig. 1A) for further functional analysis.

### Expression of *ank* genes during infection

**Figure 2:**
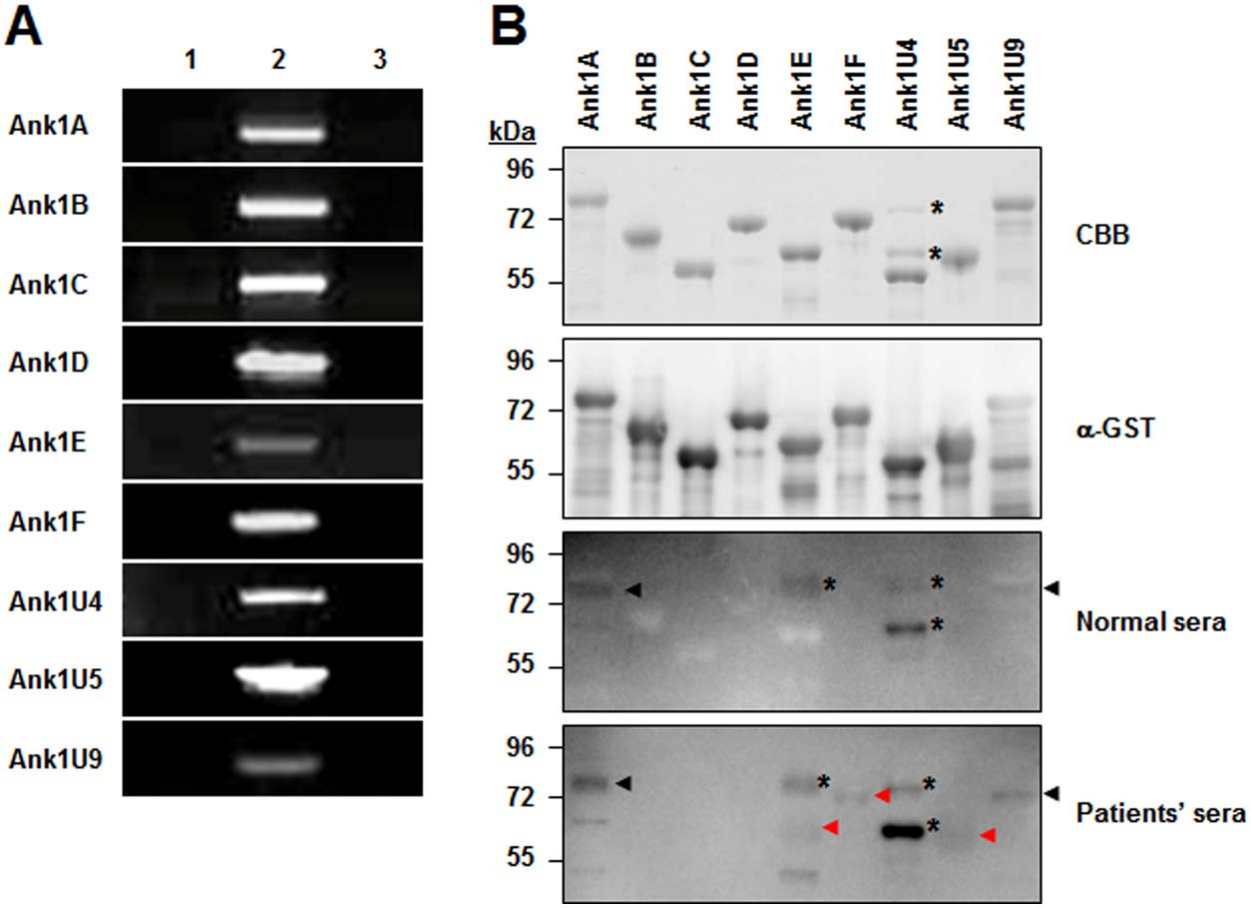
Gene expression of *ank* gene in host cells infected with *O. tsutsugamushi* and detection of recombinant Ank proteins by scrub typhus patient serum. (A) Agarose gel electrophoresis analysis of PCR products resulting from amplification of *O. tsutsugamushi* cDNA using primers (Table S1) specific to nine *ank* genes and total RNA prepared from infected L929 cells at 48 h after infection. Lane 1, cDNA from uninfected cells; lane 2, cDNA from infected cells; lane 3, total RNA from infected cells (without reverse transcription). (B) Immunoblot analysis of recombinant GST-Ank fusion proteins using anti-GST antibody or pooled sera from patients (scrub typhus-positive) or healthy volunteers (scrub typhus-negative). *, nonspecific bands with irrelevant molecular weight; black arrow, specific bands with relevant molecular weight but detected by both negative and positive sera; red arrow, specific bands with relevant molecular weight and detected only by positive sera. CBB, Coomassie brilliant blue stain.

To examine whether the selected *ank* genes are actively transcribed in *O. tsutsugamushi* during infection in mammalian host cells, we performed RT-PCR analysis using total RNA purified from infected L929 cells. To remove any contaminating oriential or host-cell genomic DNA, the extracted total RNA were treated with DNase I before use. The DNase I-treated RNA was then reverse transcribed using random primers to yield cDNA. The reverse transcription products were PCR amplified using indicated primer pairs specific to the nine selected *ank* genes (Table S1). As shown in Fig. 2A, agarose gel electrophoresis of the PCR products revealed the amplification of the oriential *ank* genes from *O. tsutsugamushi* cDNA, indicating that all nine *ank* gene transcripts were present in the total RNA of infected host cells. All PCR products were cloned and sequenced to confirm amplification of the correct gene fragments with the expected sizes. PCR products were not detectable in the control reactions using cDNA synthesized from uninfected L929 cells or total RNA from infected host cells without reverse transcription. This result demonstrates that mRNA transcripts of all nine *ank* genes were actively expressed in infected host cells.

Next, the specific antibody responses were measured, which may provide indirect evidence of Ank protein expression *in vivo*. Recombinant proteins fused with GST were purified from *E. coli* and used as antigens for immunoblot analysis using serum from scrub typhus patients and normal controls. As shown in Fig. 2B, all the purified proteins were found to react with the anti-GST antibody. To remove any potential anti-GST antibodies in the human serum, we used GST-glutathione beads to pre-clear anti-GST antibodies and confirmed the absence of reactivity against purified GST protein by immunoblot (data not shown). In the immunoblot assays using the fusion proteins, pooled serum from scrub typhus patients reacted specifically with three Ank proteins (Ank1E, Ank1F, and Ank1U5; Fig. 2B). Although two other Ank proteins (Ank1A and Ank1U9) also showed an immunoblot signal when using patient serum, they also reacted with normal human serum, suggesting the signals are non-specific. These results suggest that at least three *ank* genes are potentially expressed at the protein level during human infection and induce antibody responses. Considering that all nine *ank* genes were expressed at the transcriptional level during *in vitro* infection, some of the *ank* genes may not have been translated during *in vivo* infection or were expressed at very low levels insufficient to induce any antibody responses.

### Intracellular localization of *O. tsutsugamushi* Ank proteins in HeLa cells

**Figure 3:**
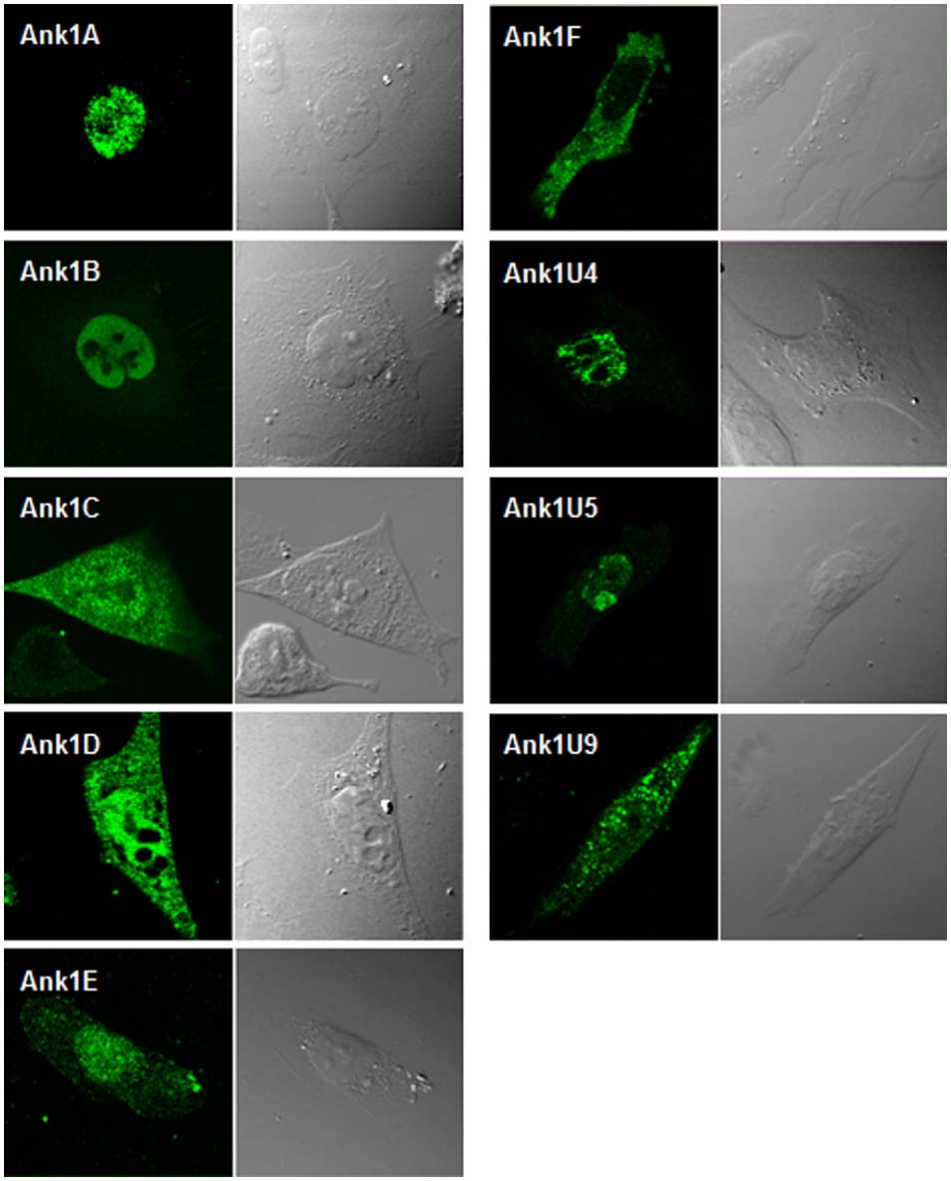
Subcellular localization of Ank proteins in HeLa cells. Nine Ank proteins were ectopically expressed in HeLa cells as N-terminal Flag-tagged forms. Cells were transfected with each construct for 18 h and subsequently fixed, permeabilized, and stained with anti-Flag antibody. Host cell nuclei can be identified in the corresponding DIC image to the right of each immunofluorescent image.

To provide insights into *O. tsutsugamushi* Ank protein function during host-cell interaction, each Ank protein was expressed in HeLa cells, and its localization was visualized by confocal fluorescence microscopy. Ank1A, -1B, -1E, -1U4, and -1U5 were mainly localized in host nuclei, whereas Ank1F was detected only in the host cytoplasm (Fig. 3). Ank1C, Ank1D, and Ank1U9 were observed in both nucleus and cytoplasm in diffuse or punctate forms. When possible localization of the Ank proteins in trafficking vesicles was examined by co-staining with endosomal and lysosomal markers, EEA1 and LAMP1 respectively, Ank proteins and the vesicular compartments were not co-localized (data not shown).

### Interaction of multiple Ank proteins with the components of the SCF1 ubiquitin ligase complex and EF1α

**Figure 4:**
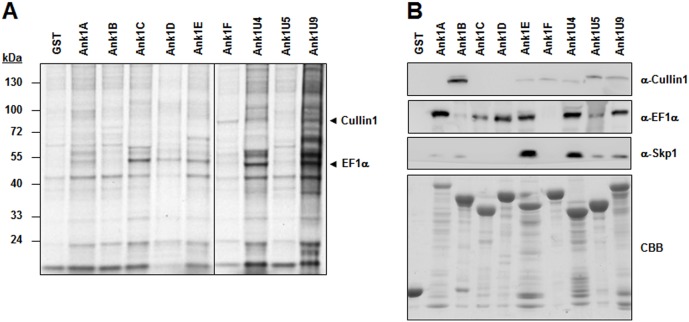
Identification of cellular proteins that interact with Ank proteins. (A) Glutathione-Sepharose beads containing GST or one of the nine Ank proteins fused with GST were mixed with ECV304 cell lysate. Cellular interacting proteins were resolved by SDS-PAGE and visualized by Coomassie brilliant blue staining. Arrows indicate Cullin1 and EF1α, which were identified by mass spectrometry. (B) Immunoblot analyses were performed using specific antibodies and the cellular protein precipitates obtained from GST pull-down assays. At the bottom of the image, GST and the recombinant Ank proteins used in the pull-down assays are visualized after Coomassie brilliant blue (CBB).

To identify host-cell proteins that interact with *O. tsutsugamushi* Ank proteins, GST pull-down assays were performed with bacterial GST fusion proteins purified from *E. coli*. Purification was performed using the cell lysate of 1×10^8^ ECV304 cells and purified GST or GST-Ank fusion proteins. Two cellular proteins specifically interacted with GST-Ank proteins but not with GST (Fig. 4). Mass spectrometry revealed that these cellular proteins were Cullin1 and EF1α. To confirm the association of Ank proteins and the cellular proteins, we performed immunoblot analysis after GST pull down. As shown in Fig. 4B, multiple Ank proteins interacted with Cullin1 as well as EF1α, whereas GST did not show any interaction. Since Cullin1 is known to be a core component of SCF1 ubiquitin ligase complexes [49], we also examined the association of Ank proteins with Skp1, which connects SCF1 with substrate-binding proteins such as F-box proteins. Immunoblot analysis using a Skp1 antibody revealed that multiple Ank proteins also interacted with Skp1 (Fig. 4B). Seven Ank proteins (Ank1A, Ank1B, Ank1E, Ank1F, Ank1U4, Ank1U5, and Ank1U9) containing F-box–like domains associated with either or both Cullin1 and Skp1, although the level of interaction with the SCF1 components was quite variable among them, which might be due to sequence variations in the F-box-like motifs (Fig. 1B). In contrast, the two Ank proteins (Ank1C and Ank1D) lacking the domain interacted only with EF1α, suggesting that the F-box–like motif may mediate the interaction of Ank proteins with SCF1. Only the Ank1F protein interacted with Cullin1 but not with EF1α. These results suggest that at least six of the nine Ank proteins may connect SCF1 with EF1α as a potential substrate for the ubiquitin ligase complex.

### Ank1U5 mediates ubiquitination and degradation of EF1α

**Figure 5:**
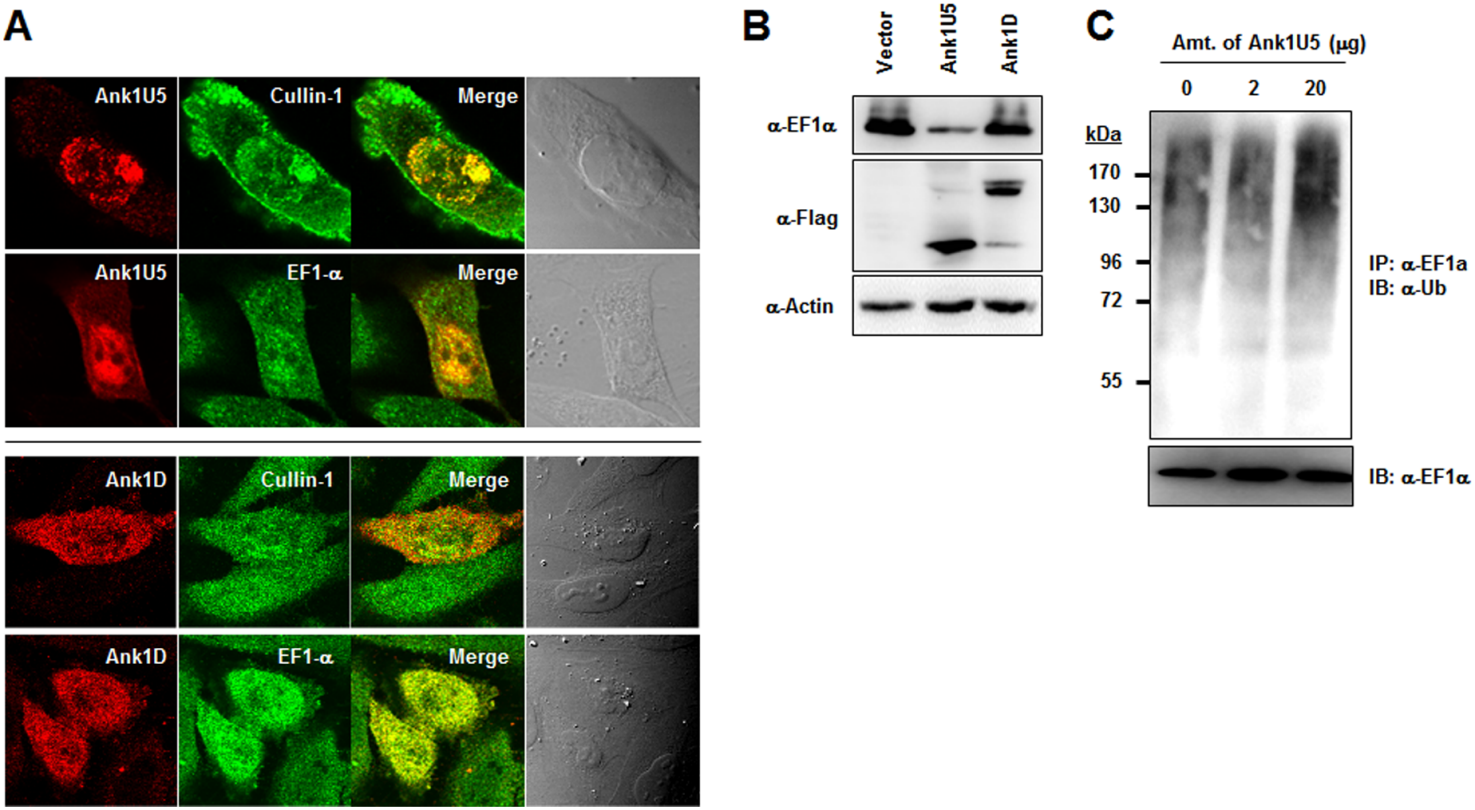
Ank1U5-mediated ubiquitination and downregulation of EF1α. (A) HeLa cells transfected with Flag-Ank1U5 for 18 h were fixed, permeabilized, and stained with anti-Flag antibody together with anti-Cullin1 or anti-EF1α antibody. Merged images show the colocalization of Ank1U5 (red) with endogenous Cullin1 and EF1α (green) in the nucleus. (B) HeLa cells transfected with either vector DNA or plasmid encoding Flag-Ank1U5 were subjected to immunoblot to monitor EF1α levels 48 h after transfection. Levels of GAPDH indicate equal protein loading. (C) The effect of Ank1U5 on EF1α ubiquitination was examined by *in vitro* ubiquitination reaction. EF1α was immunoprecipitated from the reaction mixture in the absence or presence of the recombinant GST-Ank1U5 at the indicated amounts and subjected to immunoblotting with anti-ubiquitin antibody.

To determine whether Ank proteins mediate EF1α ubiquitination, Ank1U5 was selected for the functional assays on the basis of its induction of an antibody response in scrub typhus patients and interaction with both SCF1 and EF1α. Ank1D was also examined as a control since it binds to EF1α but not to SCF1. To confirm the interaction of the Ank proteins with Cullin1 and EF1α, intracellular co-localization of the bacterial and host proteins was examined by immunofluorescence confocal microscopy after transfecting HeLa cells with a plasmid encoding Ank1U5 or Ank1D. As shown in Fig. 5A, Ank1U5 co-localized with endogenous Cullin1 and EF1α mainly in the host nuclei. In Ank1U5-expressing cells, EF1α was efficiently recruited to the nucleus where it co-localized with Ank1U5 (Fig. 5A, upper panels). Ank1D also co-localized with endogenous EF1α throughout the cytoplasm and nucleus, whereas it was partially co-stained with Cullin1 and their co-localization was much less prominent than EF1α (Fig. 5A, lower panels).

To assess the potential degradation of EF1α via ubiquitination-dependent pathways, the EF1α protein level was next investigated in cells expressing Ank1U5 or Ank1D. Equal amounts of protein from HeLa cells transfected with a vector or plasmid encoding Ank1U5, or Ank1D were subjected to immunoblot assay. The protein level of EF1α was dramatically reduced in cells expressing Ank1U5 compared to that of control cells (Fig. 5B), whereas there was no detectable change of EF1α level in cells expressing Ank1D. These results strongly suggest that specific downregulation of EF1α by the Ank protein requires the interaction with SCF1 ubiquitin ligase complex. Finally, the possible mediation of EF1α ubiquitination by Ank1U5 was investigated. EF1α was immunoprecipitated from an *in vitro* ubiquitination reaction mixture in the presence of bacterially expressed GST-Ank1U5. By immunoblotting with anti-ubiquitin antibody, bands of large molecular size were detected (Fig. 5C), indicating that EF1α was ubiquitinated. High-molecular–weight ubiquitinated products increased as increasing amounts of GST-Ank1U5 were added to the reaction mixture. These results clearly demonstrate that Ank1U5 mediated the degradation of EF1α by enhancing the polyubiquitination of the host protein.

### Degradation of EF1α in host cells infected with *O. tsutsugamushi*

**Figure 6:**
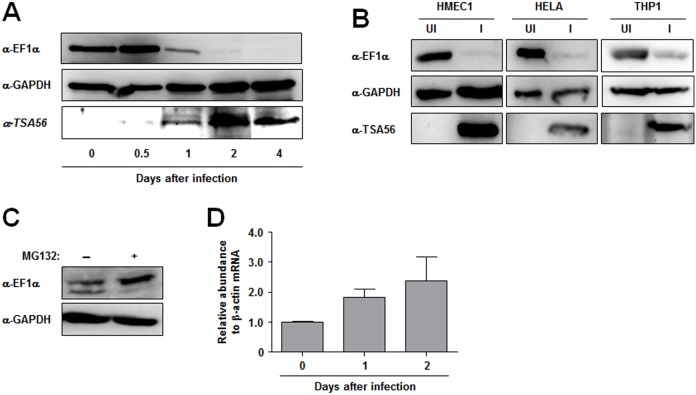
Downregulation of EF1α in various types of host cell infected with *O. tsutsugamushi*. (A) The results of immunoblot analysis of the total cellular protein isolated from ECV304 cells infected with *O. tsutsugamushi* at the indicated time points are shown. Protein levels were standardized to GAPDH, and the level of TSA56, a type-specific antigen of *O. tsutsugamushi*, was monitored to confirm bacterial replication during the infection periods. EF1α levels during the infection were analyzed using anti-EF1α antibody. (B) EF1α levels were analyzed in lysates prepared from different types of cell lines at 2 d after the infection with *O. tsutsugamushi* and compared with that of uninfected cells. GAPDH and TSA56 were monitored simultaneously as protein loading and bacterial infection controls, respectively. UI, uninfected; I, infected. (C) EF1α levels were analyzed in HeLa cells treated with MG132 (10 µM) for 4 h at 2 d after infection with *O. tsutsugamushi*. GAPDH was used as protein loading control. (D) EF1α mRNA levels were examined by real-time RT-PCR and normalized to β-actin mRNAs in HeLa cells infected with *O. tsutsugamushi*. The data are presented as mean+SD of three independent experiments.

To explore whether the level of EF1α in host cells was also influenced by infection with *O. tsutsugamushi*, the kinetics of EF1α stability after bacterial infection were examined. ECV304 cells were infected with *O. tsutsugamushi*, and cell extracts were harvested at different time points after bacterial infection to monitor endogenous EF1α using an anti-EF1α antibody. As shown in Fig. 6A, bacterial infection decreased the level of EF1α dramatically in a time-dependent manner. 2 days after the infection, EF1α was almost undetectable by immunoblot assay, whereas the level of a control housekeeping protein, GAPDH, was not significantly changed during infection, indicating that the degradation of EF1α was a specific response to *O. tsutsugamushi* infection. The reduction of EF1α was further verified in different types of host cells; endothelial (HMEC1), epithelial (HeLa), and monocytic (THP1) cells were examined. 2 days after infection, the level of EF1α was dramatically reduced in all cell types, as shown in Fig. 6B. To further confirm the proteasomal degradation of EF1α, *O. tsutsugamushi*-infected HeLa cells were treated with proteasome inhibitor, MG132, for 4 h at 2 days after infection. Fig. 6C shows that cells treated MG132 accumulated significantly higher amounts of EF1α compared to untreated cells. When we measured mRNA level for EF1α by real time RT-PCR, EF1α transcripts slightly increased in infected cells up to 2 days after infection (Fig. 6D), indicating that EF1α protein levels were mainly regulated by post-translational control during the *O. tsutsugamushi* infection. Taken together, these results suggest that *O. tsutsugamushi* infection leads to a specific reduction of EF1α, potentially via secreted Ank proteins, via ubiquitination-dependent proteasomal degradation.

## Discussion

The *O. tsutsugamushi* genome has an extraordinary structure, with up to 40% comprised of repeat sequences in the form of mobile genetic elements and accessory genes that are putatively involved in host-parasite interactions [22]. Acquisition and extensive proliferation of mobile genetic elements, including conjugative T4SS, together with genes encoding eukaryotic-like proteins such as Ank proteins may have resulted from long-term adaptation processes in isolated intracellular niches, where selective constraints acting on intracellular bacteria influence their ability to invade and exploit host cells for survival and replication [24]. In this study, we investigated the potential role of *O. tsutsugamushi* Ank proteins in host-pathogen interactions.

As a first step, *ank* gene expression was examined within host cells infected with *O. tsutsugamushi*. All nine selected genes were detected at the transcriptional level (Fig. 2A). This result is partially consistent with our previous report, in which the expression of several *ank* genes was observed in a microarray-based transcriptome analysis, but no Ank proteins were detected by proteome analysis of the whole bacterial lysate [27]. In the current study, specific but weak antibody responses against only three of nine Ank proteins tested were detected using serum from scrub typhus patients (Fig. 2B). This finding may be the result of very low levels of *O. tsutsugamushi* Ank protein expression, even *in vivo*, or weak immunogens that are insufficient to induce strong antibody responses. In addition, Ank protein expression might be differentially controlled within different host systems, as reported in a previous study in which at least two Ank proteins were upregulated in macrophages compared to fibroblasts [27]. Previously, some Ank proteins expressed in *C. burnetti* [50] and *Ehrlichia* sp. [51] were found to react strongly with immune sera and could be utilized as target antigens for serodiagnosis. Considering our results, *O. tsutsugamushi* Ank proteins may not be an effective target for this purpose. Nevertheless, we detected active transcription of several *ank* genes in *O. tsutsugamushi*-infected cells and specific antibody responses in human scrub typhus patients, indicating that Ank proteins might be expressed in mammalian host cells after *O. tsutsugamushi* infection.

Accumulating evidence suggests that Ank proteins found in several intracellular bacteria might play a critical role in host-pathogen interactions for bacterial survival and pathogenesis [32]. Interactions between intracellular pathogens and their eukaryotic hosts were found to be mediated in part by the secretion of Ank proteins into host cells [32]. Three *L. pneumophila* Ank proteins were required for intracellular proliferation within human macrophages and protozoa after translocation through T4SS [52], [53]. Moreover, the *L. pneumophila* AnkX protein directly mediates the covalent attachment of a phosphocholine moiety to Rab1 and Rab35, which direct the transport and assembly of the membrane-bound compartment in which *L. penumopnila* resides [54]. *A. phagocytophilum* AnkA protein was found to translocate into host cells via the T4SS, where it localized to the cytosol and nucleus and was phosphorylated by Abl-1 to facilitate infection [41]. AnkA protein also bound to nuclear proteins and formed complexes with AT-rich DNA sequences, resulting in the modulation of host gene transcription [55], [56]. The *E. chaffeensis* p200 protein, which contains 21 Ank repeats, translocated to the nuclei of infected monocytes and interacted with an adenine-rich motif of Alu-Sx elements located in the promoters and introns of various host genes [57], suggesting that p200 may also affect gene expression globally in host cells. In *C. burnetti*, 11 Ank proteins are translocated into the host cytosol in a T4SS-dependent manner when using *L. pneumophila* as a surrogate host [33]. Ectopically expressed Ank proteins are localized to a variety of subcellular regions including microtubules, mitochondria and parasitophorous vacuoles in mammalian cells, suggesting that they modulate diverse host functions [33].

Although the presence and conservation of T4SS and prevalence of *ank* genes in the *O. tsutsugamushi* genome [22], [43], [58] strongly suggest that Ank proteins may play a critical role in modulating host-cell processes during *O. tsutsugamushi* infection, translocation of Ank proteins found in Rickettsiacea members, *Rickettsia* and *Orientia*, is poorly characterized to date. Ank proteins in the bacterial order, Rickettsial Ankyrin repeat protein 1 (RARP-1) of *R. typhi* and Ank200 of *E. chaffeensis,* were recently shown to be secreted via T1SS [38], [39]. Since genetic modification of *O. tsutsugamushi* is still impossible, it is hard to determine whether *O. tsutsugamushi* Ank proteins are secreted via a specific bacterial secretion system.

Secreted bacterial Ank proteins often traffic to distinct cellular locations, where they modulate specific host-cell functions during infection [33], [40], [41], [57]. Ectopic expression of *O. tsutsugamushi* Ank proteins in HeLa cells demonstrated that the potential effectors are consistently observed in specific subcellular compartments, the nucleus and/or cytoplasm, in diffuse or punctate forms (Fig. 3). The differential patterns of subcellular localization suggest that these effectors may regulate diverse host functions in specific cellular compartments, as described previously [32]. However, it is interesting to note that six of nine Ank proteins were preferentially localized to the nucleus, while only one was exclusively detected in the cytoplasm. Thus, multiple *O. tsutsugamushi* Ank proteins could be postulated to function preferentially in the nucleus to modulate host cells or play redundant roles by targeting the same host factor. This hypothesis is further supported by the findings that many of the *O. tsutsugamushi* Ank proteins share conserved domain structures (Fig. 1A) and the same cellular target proteins (Fig. 5). A domain search of the Ank proteins detected F-box–like domains (or PRANC domains) in addition to Ank domains in the C-termini of at least seven proteins.

In the Pfam database, approximately 330 proteins were identified in all taxa that contained PRANC domains. Most of the genes encoding PRANC proteins shared the domain structures (N-terminal Ank domains and C-terminal PRANC domain) and were found in a variety of poxvirus families. Only 24 genes were detected in the bacterial kingdom. Among these 24 bacterial proteins, 20 proteins are encoded by *O. tsutsugamushi* (strain Boryong or Ikeda) [22], [23], and four proteins by *Wolbachia* sp. and *Rickettsiella grylli*. Amino acid sequence analysis revealed that the C-terminal PRANC domain in the poxviral and rickettsial proteins contains the most conserved amino acids of the eukarytotic cellular F-box domain (Fig. 1B). Even though the viral and bacterial PRANC domain is shorter than the cellular F-box domain which is typically located in the N-terminus [48], [49], the structural features of viral and bacterial Ank-PRANC proteins suggest that the protein family may function as a target recognition subunit, and thus have the same function as cellular F-box proteins.

Multiple pox viral PRANC proteins have been reported to interact with core components of the cellular SCF1 ubiquitin ligase [49], [59], an E3 ubiquitin ligase complex that recruits target proteins via F-box proteins and ubiquitinates the protein substrate for degradation by the proteasome [60]. For example, an Ank-PRANC protein, M-T5, from a rabbit-specific Myxoma virus was shown to regulate viral tropism within rabbit lymphocytes and some classes of human cancer cell lines [61]. M-T5 co-localized with host-cell Cullin1 in the nucleus [61] and functioned as a virus-encoded cell cycle regulator by promoting the phosphorylation, ubiquitination, and degradation of p27/Kip1 [62]. Unlike M-T5, however, specific host-binding partners or target substrates have not yet been identified for most of the poxviral Ank-PRANC proteins, except for SCF complexes [63].

In this study, at least six *O. tsutsugamushi* Ank-PRANC proteins were found to interact with both SCF components and EF1α (Fig. 4). Considering the results from poxviral Ank-PRANC proteins, the C-terminal PRANC domain may mediate interaction with the SCF complex, whereas the N-terminal Ank domain may bind to cellular EF1α. This possibility is further supported by the findings that two Ank proteins (Ank1C and Ank1D) lacking PRANC domains failed to interact with SCF components and that eight Ank proteins (all except Ank1F) specifically bound to EF1α. Ank1C, which is largely comprised of Ank domains only, also interacted with EF1α. Therefore, the Ank domains of the *O. tsutsugamushi* proteins may be redundant in function for targeting cellular EF1α. In addition, one of the Ank-PRANC proteins, Ank1U5, was co-localized specifically with Cullin1 and EF1α within host nuclei and capable of downregulating the level of EF1α, potentially by enhanced ubiquitination, whereas Ank1D, interacting with EF1α but not with Cullin1, failed to induce downregulation of EF1α (Fig. 5). The downregulation of EF1α was consistently observed in diverse host cell types infected with *O. tsutsugamushi* (Fig. 6).

What is the biological role of these Ank-PRANC domain proteins, especially with regard to the interaction and downregulation of EF1α? EF1α has been well-established as responsible for loading charged amino acids to the A site of the ribosome during protein synthesis. Recently, TPR32 of *E. chaffeensis* was shown to interact with multiple host proteins including EF1α and may affect intracellular survival within a host cell [64]. In addition, four *L. pneumophila* bacterial virulence factors, Lgt, Sidl, Lgt2, and Lgt3, were shown to interact with EF1α and inhibit host protein synthesis [65]–[67]. When host protein synthesis was examined in cells expressing *O. tsutsugamushi* Ank proteins or infected with *O. tsutsugamushi*, however, no significant effect on eukaryotic protein translation was observed (data not shown). Previously, it was also shown that cellular levels of EF1α, constituting 1∼2% of total cellular proteins, are not rate limiting for protein synthesis [68], [69]. Therefore, *O. tsutsugamushi* Ank proteins are likely to regulate other cellular processes by downregulating EF1α in infected host cells. Although the canonical role of EF1α in eukaryotic translation is associated with a cytoplasmic function, a number of recent works support the hypothesis that EF1α is a central regulator involved in the coordination of many different cellular processes [70], including the nuclear export of proteins from the nucleus [71], protein quality control and co-translational degradation [72], cytoskeletal regulation [73], and control of cellular apoptosis [74]. Although it remains to be demonstrated whether *O. tsutsugamushi* Ank proteins perturb various cellular processes by downregulating host EF1α, conserved targeting and subsequent degradation of EF1α by multiple *O. tsutsugamushi* Ank proteins via linkage to the SCF ubiquitin ligase complex could be a novel strategy for bacterial replication and/or pathogenesis during mammalian host infection.

## Supporting Information

Table S1 **Primers used in this study.**
(DOC)
