# Supplementary material for: Multiple Orientia tsutsugamushi Ankyrin Repeat Proteins Interact with SCF1 Ubiquitin Ligase Complex and Eukaryotic Elongation Factor 1 α
Source: PLoS One. 2014 Aug 28;9(8):e105652. doi: 10.1371/journal.pone.0105652 (PMC4148323; doi:10.1371/journal.pone.0105652)
Supplement: Table S1 — Primers used in this study. (DOC) [file pone.0105652.s001.doc]

**Supporting information**

**Table S1. Primers used in this study**

| **Primer name and use** | | **Sequence ^a^** |
| --- | --- | --- |
| *Primers for RT-PCR (product size)* | | |
| Ank1A (402 bp) | *Forward* 5'- GCTGCAGAATGCGGTAATACAAAG -3'  *Reverse* 5'- CAAAGCAGTCTTTCCCCAAATATCC -3' | |
| Ank1B (422 bp) | *Forward* 5'- GGTCTGATGGTAATTATCCAGACGC -3'  *Reverse* 5'- GCTTTCGCAAACAATTGTGTCTTTC -3' | |
| Ank1C (396 bp) | *Forward* 5'- GTGGACACACGGCTTTACATACAG -3'  *Reverse* 5'- CTATATGATCATCGCAATAGGGAGG -3' | |
| Ank1D (450 bp) | *Forward* 5'- GCTGATCCCAATGTACCATCATATG -3'  *Reverse* 5'- CCTTTCATCCTTACTGAAGCCCC -3' | |
| Ank1E (343 bp) | *Forward* 5'- CCTTGGTTGCTTATACTGTTAAGC -3'  *Reverse* 5'- GATTCAACTGCTCCTTGTAGCAGC -3' | |
| Ank1F (322 bp) | *Forward* 5'- CATTACGCTACAAACAGTTTTCACC -3'  *Reverse* 5'- CAGCAGCATGATGTAACGGAGTGC -3' | |
| Ank1U4 (436 bp) | *Forward* 5'- CGTCTTCTGTTAAATCATGGACCTG -3'  *Reverse* 5'- CTTTTCCTTCTTCAACAGCTTCTTC -3' | |
| Ank1U5 (405 bp) | *Forward* 5'- GCTTCTATTGAAGCATGGAGCTG -3'  *Reverse* 5'- GATAAGCCACTTTCGTTAACGTAGG -3' | |
| Ank1U9 (343 bp) | *Forward* 5'- GGTATGAACGTTGTACATTGCGCTG -3'  *Reverse* 5'- CAGCAAAATGCATAACTGTGTTACC -3' | |
| hEF1A | *Forward* 5'- GAAGACCCACATCAACATCGT -3’  *Reverse* 5'- CTCCGCATTTGTAGATGAGGTG -3’ | |
| β-Actin | *Forward* 5'- CATGTACGTTGCTATCCAGGC -3’  *Reverse* 5'- CTCCTTAATGTCACGCACGAT -3’ | |
| *Primers for pGEX4T-1 cloning* | | |
| Ank1A | *Forward 5'- GGCGAATTCATGAAAAATTGTCTTTCTTGGATATATAG -3'*  *Reverse 5'- GGCCTCGAGTTAGTTTTCTATATCAACTACTTCTTCTCC -3'* | |
| Ank1B | *Forward 5'- GGCGAATTCATGTATAATACTGATTTACATGATGCTGC -3'*  *Reverse 5'- GGCCTCGAGCTACGATTCTTCATGCATAACATTATTGC -3'* | |
| Ank1C | *Forward 5'- GGCGAATTCATGTCTGAATCTAAGGCTAAATATGCTTTAC -3'*  *Reverse 5'- GGCCTCGAGCTAAGACCATGGAACTGATTGTTGCGTTAG -3'* | |
| Ank1D | *Forward 5'- GGCGGATCCATGAATAACGGTAATTTATTACATGAATTATATC -3'*  *Reverse 5'- GGCCTCGAGTTATGAATTAGTACAGCCTCCCATAACTTC -3'* | |
| Ank1E | *Forward 5'- GGCGAATTCTTGTATTATGCTGTTGCAAAAGGATACATAG -3'*  *Reverse 5'- GGCGCGGCCGCCTACTCTCCCTCATATATGGCATGTG -3'* | |
| Ank1F | *Forward 5'- GGCGGATCCATGAATACTGCTTTGAGTTTTGCTATTAAGCG -3'*  *Reverse 5'- GGCCTCGAGTTATATGCATCTGTGACCTGAAGGCTGAGGATC -3'* | |
| Ank1U4 | *Forward 5'- GGCGAATTCATGTATAAGTTTTTACCGCTTCGTGC -3'*  *Reverse 5'- GGCCTCGAGTTATTCTTTCTCATATATAGCATGTGCTCC -3'* | |
| Ank1U5 | *Forward 5'- GGCGAATTCATGTGTAATACTGCTTTACATGAAGCTG -3'*  *Reverse 5'- GGCCTCGAGCTATTCTCCCTCATATAGAGCATATGCTC -3'* | |
| Ank1U9 | *Forward 5'- GGCGAATTCATGGATCAGTTATTACGTTATATATTATTAC -3'*  *Reverse 5'- GGCCTCGAGTTAGACTGAACTCTCGTTACCTACACC -3'* | |
| *Primers for p3xFlagCMV10 cloning* | | |
| Ank1A | *Forward 5'- GGCGAATTCAATGAAAAATTGTCTTTCTTGGATATATAG -3'*  *Reverse 5'- GGCGCGGCCGCTCAGTTTTCTATATCAACTACTTCTTCTCC -3'* | |
| Ank1B | *Forward 5'- GGCGAATTCAATGTATAATACTGATTTACATGATGCTGC -3'*  *Reverse 5'- GGCGGATCCCTACGATTCTTCATGCATAACATTATTG -3'* | |
| Ank1C | *Forward 5'- GGCGAATTCAATGTCTGAATCTAAGGCTAAATATGC -3'*  *Reverse 5'- GGCGGATCCCTAAGACCATGGAACTGATTGTTGCG -3'* | |
| Ank1D | *Forward 5'- GGCGCGGCCGCGATGAATAACGGTAATTTATTACATGAATTA -3'*  *Reverse 5'- GGCGGATCCTCATGAATTAGTACAGCCTCCCATAAC -3'* | |
| Ank1E | *Forward 5'- GGCGAATTCATTGTATTATGCTGTTGCAAAAGGATACATAG -3'*  *Reverse 5'- GGCGGATCCCTACTCTCCCTCATATATGGCATGTG -3'* | |
| Ank1F | *Forward 5'- GGCGCGGCCGCGATGAATACTGCTTTGAGTTTTGCTATTAAGC -3'*  *Reverse 5'- GGCGGATCCTTATATGCATCTGTGACCTGAAGGC -3'* | |
| Ank1U4 | *Forward 5'- GGCGAATTCGATGTATAAGTTTTTACCGCTTCGTGC -3'*  *Reverse 5'- GGCGGATCCCTATTCTTTCTCATATATAGCATGTGC -3'* | |
| Ank1U5 | *Forward 5'- GGCGAATTCGATGTGTAATACTGCTTTACATGAAGCTG -3'*  *Reverse 5'- GGCTCTAGACTATTCTCCCTCATATAGAGCATATGCTC -3'* | |
| Ank1U9 | *Forward 5'- GGCGAATTCAATGGATCAGTTATTACGTTATATATTATTACATTG -3'*  *Reverse 5'- GGCGGATCCTTAGACTGAACTCTCGTTACCTAC -3'* | |

**^a^** Underlining indicates restriction sites in primers.
